# Diagnostic value of phenotypic testing combined with molecular biology testing for tuberculosis

**DOI:** 10.1038/s41598-026-43218-z

**Published:** 2026-03-06

**Authors:** Xianlei Wang, Cong He, Ming Liu, Huan Zhang, Jiong Xie, Xuefeng Zhao, Aihong Meng

**Affiliations:** 1 Department of Respiratory and Critical Care Medicine, The Second Hospital of Hebei Medical University, Hebei, China; 2 Department of Tuberculosis, Hebei Chest Hospital, Hebei, China; 3 Department of Intensive Care Unit, Hebei General Hospital, Hebei, China; 4 Department of Respiratory and Critical Care Medicine, Shijiazhuang People’s Hospital, Hebei, China; 5 Hebei Key Laboratory of Pulmonary Diseases, Shijiazhuang, Hebei China

**Keywords:** Tuberculosis, Liquid-based sandwich cup, Mycobacterial culture, Boao MTB TaqMan-qPCR, GeneXpert MTB/RIF, Diseases, Medical research, Molecular medicine

## Abstract

To evaluate the diagnostic value of combining phenotypic testing with molecular biology testing for tuberculosis, a retrospective study was conducted on 264 presumed pulmonary tuberculosis patients across four hospitals from July 2021 to September 2023. The area under the receiver operating characteristic curve (AUROC), sensitivity, specificity, positive predictive value and negative predictive value were calculated for two phenotypic testing methods—liquid-based sandwich cup acid-fast staining and mycobacterial culture—and two molecular biology testing methods—Boao MTB TaqMan-qPCR and GeneXpert MTB/RIF—using sputum and bronchoalveolar lavage fluid specimens for the diagnosis of active pulmonary tuberculosis. Using the final clinical diagnosis as the reference standard, the combination of both two specimen types and all four testing methods yielded the highest AUROC of 0.917 and sensitivity of 83.3%, significantly outperforming either multiple specimens tested by a single method or multiple methods applied to a single specimen. Moreover, combining multiple tests on either sputum or BALF specimens provided superior diagnostic performance compared with individual tests alone. This study demonstrates that integrating phenotypic and molecular biology testing, across multiple specimen types can provide more accurate guidance for the early diagnosis and treatment of pulmonary tuberculosis.

**Supplementary Information:**

The online version contains supplementary material available at 10.1038/s41598-026-43218-z.

## Introduction

The global burden of tuberculosis (TB) has risen dramatically, making it the deadliest infectious disease worldwide^1,2^. The urgency for enhanced TB prevention and control measures is therefore greater than ever, presenting a serious public health challenge^3^. Early and rapid diagnosis is essential for timely treatment and is a key determinant influencing TB control^4–7^. Advances in diagnostic technologies and procedures, such as bronchoscopy, have markedly improved the detection rates of microbiological positive cases^8^.

TB confirmation primarily relies on microbiological testing methods; however, traditional acid-fast bacilli smear microscopy has a low positivity rate, and mycobacterial culture takes 2 to 3 weeks or longer^9,10^. Immunological testing methods, including the tuberculin skin test, new EC skin tests, and interferon-gamma release assays, are ineffective in differentiating between latent TB infection and active disease^11^. Currently employed phenotypic methods include liquid-based sandwich cup (LBSC) acid-fast staining^12,13^ and mycobacterial culture^14,15^. Meanwhile, recent developments in molecular biology have led to the increased application of various molecular diagnostic techniques for TB, such as real-time PCR assays targeting mycobacterial DNA (e.g., Boao MTB TaqMan-qPCR), semi-nested real-time fluorescence quantitative PCR (e.g., GeneXpert MTB/RIF), loop-mediated isothermal amplification, and RNA-based assays^16–19^.

Although LBSC has improved positivity rates compared with conventional smear microscopy, it cannot differentiate between *Mycobacterium* tuberculosis and non-tuberculous mycobacteria (NTM) or distinguish viable from nonviable bacilli^20^. Obtaining pathological tissue samples also remains challenging. DNA-based detection methods for *Mycobacterium* tuberculosis are rapid and sensitive but are prone to false positives^21^ and likewise cannot differentiate between dead and live bacteria^22,23^. Furthermore, many patients produce scant or no sputum, and some are unable to expectorate at all. Clinically, it is often observed that patients with negative sputum tests may yield positive bronchoalveolar lavage fluid (BALF) smears^24^. Many patients present with scant or no sputum, and some may have difficulty producing sputum. Clinically, it is often observed that some patients with negative sputum tests have positive bronchoalveolar lavage fluid smears^25^. Furthermore, performing sputum tests immediately after bronchoscopy can significantly enhance sputum positivity rates. In addition, performing sputum tests immediately after bronchoscopy can significantly increase sputum positivity rates.

Taken together, these challenges underscore the value of integrating molecular and microbiological testing with bronchoscopy. Such a multimodal strategy holds promise for enhancing the detection of *Mycobacterium* tuberculosis, assessing disease activity, and differentiating TB from NTM infections. The aim of this study was therefore to evaluate a combined approach using both phenotypic and molecular methods, with the expectation that this strategy will improve the clinical diagnosis of pulmonary tuberculosis (PTB), increase detection rates, and facilitate the distinction between TB and NTM infections.

## Materials and methods

### Study population and clinical diagnosis

We retrospectively reviewed the clinical and laboratory testing results of patients with presumed PTB who provided sputum or BALF specimens at Hebei Chest Hospital, The Second Hospital of Hebei Medical University, Hebei General Hospital and Shijiazhuang People’s Hospital from July 2021 to September 2023. Medical data were collected, including age, sex, comorbidities, computed tomography imaging characteristics and final clinical diagnosis. After data collection, desensitization procedures were applied to ensure no identifying information was available for individual participants. The inclusion criteria were as follows: (1) hospitalized patients presumed of having PTB, aged 15 years or older, regardless of gender, (2) patients who underwent testing with sputum or BALF specimens, and (3) availability of a definitive final clinical diagnosis. Patients who did not meet any of these criteria were excluded. The diagnostic performance of each method was evaluated using the final clinical diagnosis as the reference standard, which was established by integrating clinical manifestations, imaging findings, immunological, microbiological and pathological evidence, and further validated by sustained clinical response during anti-tuberculosis treatment with follow-up for one year after treatment completion.

### Specimen collection

*Sputum specimens*: Sputum from the lower respiratory tract was collected using sterile cups with spiral-sealed lids within 24 h of hospital admission. A minimum volume of 10 mL of deep-coughed sputum was required. The samples were homogenized and subsequently aliquoted in a biosafety cabinet. Sample quality was assessed microscopically according to established criteria: fewer than 10 squamous epithelial cells per low-power field, more than 25 white blood cells per field, or a WBC-to-epithelial cell ratio greater than 2.5^26^. 

*BALF Specimens*: A negative pressure system was utilized to connect to the collection container. The optimal lavage site was selected based on the location of the lesions. Fifty milliliters of 0.9% sterile saline at 37 °C was instilled at the lesion opening, followed by immediate suction at a negative pressure of 50 mmHg to recover the BALF. The recovery rate for the lavage fluid was set at ≥ 30%.

### Test methods

*Phenotypic Test*: (1) Liquid-Based Sandwich Cup Acid-Fast Staining: Specimens are treated with a digestive solution and filtered through a membrane-layered cup, concentrating acid-fast bacilli on a nanostructured silica membrane. The membrane is stained via the Ziehl-Neelsen method and examined microscopically. (2) Mycobacterial culture: Specimens were inoculated into the BD BACTEC MGIT 960 liquid culture system, which automatically monitors mycobacterial growth. Positive cultures were confirmed by Ziehl–Neelsen staining, with further identification of Mycobacterium tuberculosis complex when required. Contaminated cultures were excluded after subculture on conventional media.

*Molecular Biology Test*: (1) Boao MTB TaqMan-qPCR: This method uses dual real-time fluorescence PCR and TaqMan probe technology for quantitative detection of mycobacterial nucleic acids. (2) GeneXpert MTB/RIF: A fully automated method that integrates real-time quantitative PCR for rapid diagnosis of tuberculosis and multidrug-resistant tuberculosis, covering specimen processing, DNA extraction, nucleic acid amplification, and specific detection of *Mycobacterium* tuberculosis and rifampicin resistance mutations.

All phenotypic and molecular assays were performed in the Hebei Key Laboratory of Pulmonary Diseases, an officially accredited provincial-level research laboratory certified by the Hebei Provincial Department of Science and Technology. The laboratory operates under standardized management and quality control procedures consistent with ISO 15,189 requirements to ensure the reliability and reproducibility of test results.

### Statistical analysis

All information of patients enrolled in the study was recorded in Microsoft Excel, and all statistical analyses were performed in Python 3.10.0. The values and 95% confidence intervals (95%CI) of areas under the receiver operating characteristic curve (AUROC), sensitivity, specificity, positive predictive value (PPV) and negative predictive value (NPV) were calculated across 1000 bootstrap replications for all samples. The two-tailed DeLong test followed by Bonferroni test for multiple comparisons was used to compare the AUCs of derived methods^27^. Moreover, groups were compared using the independent two-tailed t-test for continuous variables with normal distribution, the two-tailed Wilcoxon rank sum test for variables with non-normal distribution and the Chi-square test for categorical variables. Statistically significant difference was considered at *p* < 0.05. Given the retrospective design, no a priori sample size calculation was performed; instead, the adequacy of the sample size was evaluated based on the precision of estimated diagnostic performance metrics and their bootstrap-derived 95% confidence intervals.

## Results

### Patients and study collective characteristics

**Fig. 1:**
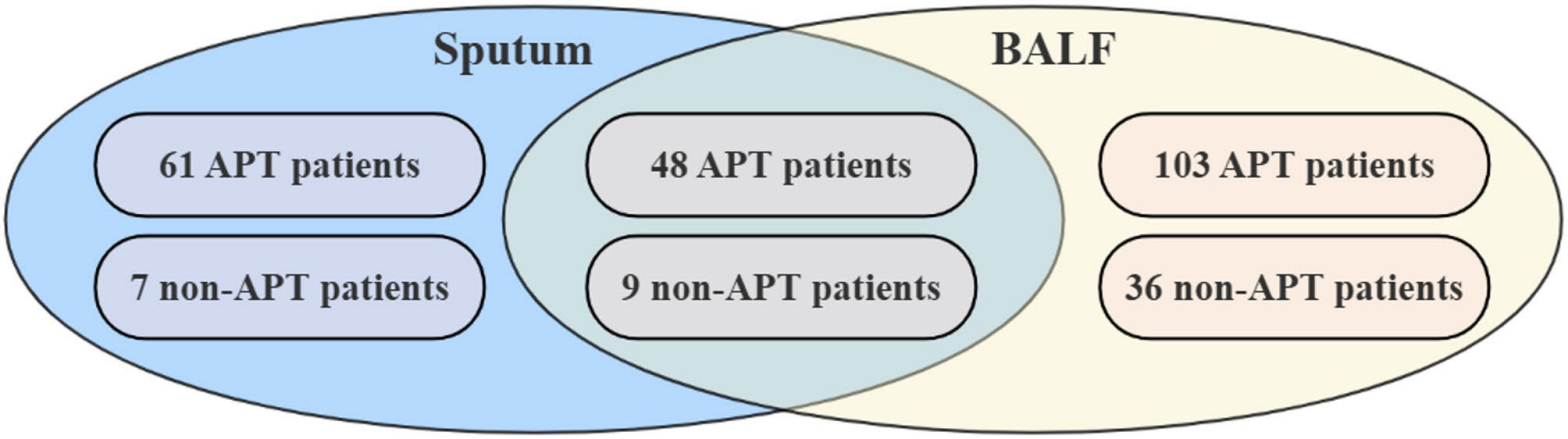
Venn diagram of data overlap among study groups.

According to the national diagnostic criteria (WS 288–2017 for tuberculosis)^28^, 264 clinically diagnosed patients were enrolled in this study. Inclusion required submission of at least one sputum or BALF specimen, and all collected specimens were analyzed with the four diagnostic methods described above. In routine clinical practice, repeated sputum smear examinations were performed whenever possible, and BALF specimens were additionally tested in patients undergoing bronchoscopy, thereby reducing the risk of false-negative smear results. The detailed data overlap among the study groups is shown in Fig. 1.

Demographic and clinical characteristics of the study population were presented in Table 1. Of the enrolled patients, 167 (63.3%) were males, with a median age of 50 years (interquartile range [IQR]: 29–64). 46 patients (17.4%) were diagnosed with diabetes mellitus. 190 patients (72.0%) presented with cough, while 145 patients (54.9%) reported expectoration. Additionally, CT imaging revealed effusion in 114 patients (43.2%) and calcification in 128 patients (48.5%). Detailed comparisons of the relevant characteristics between patients with and without active pulmonary tuberculosis (APT) are presented in Supplementary Materials (Sect. 2).

**Table 1:** Characteristic of the study population.

Characteristics	Total (*n* = 264)	APT (*n* = 212)	non-APT (*n* = 52)	*p* value
Age [Median (IQR)]	50 (29, 64)	44 (26, 63)	58 (46, 66)	0.022
Gender/Male [n (%)]	167 (63.3)	138 (65.1)	29 (55.8)	0.276
Diabetes Mellitus [n (%)]	46 (17.4)	39 (18.4)	7 (13.5)	0.524
Cough [n (%)]	190 (72.0)	157 (74.1)	33 (63.5)	0.176
Expectoration [n (%)]	145 (54.9)	122 (57.5)	23 (44.2)	0.116
Computed Tomography Imaging				
Effusion [n (%)]	114 (43.2)	96 (45.3)	18 (34.6)	0.217
Calcification [n (%)]	128 (48.5)	106 (50.0)	22 (42.3)	0.401

*P*-values were obtained by comparing the characteristics between APT and non-APT patients.

### Clinical performance by combining multiple testing methods on sputum specimens

**Fig. 2:**
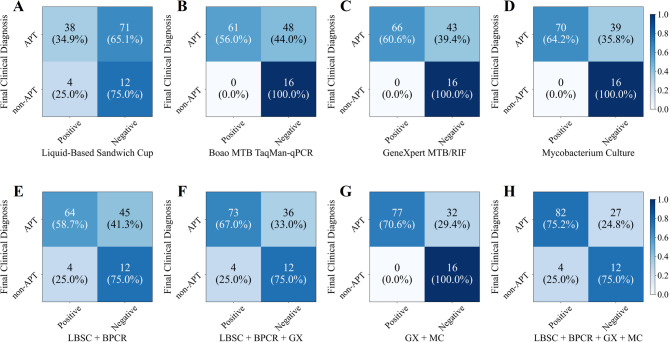
Confusion matrix of multiple testing methods on sputum specimens.

For the analysis of sputum specimens, 125 patients were selected from the study population, each undergoing LBSC, Boao MTB TaqMan-qPCR, GeneXpert MTB/RIF and mycobacterial culture. The results revealed that 42 patients (33.6%) tested positive for LBSC, 61patients (48.8%) tested positive for Boao MTB TaqMan-qPCR, 66 patients (52.8%) tested positive for GeneXpert MTB/RIF, and 70 patients (56.0%) tested positive for mycobacterial culture (Fig. 2). Additionally, the combined diagnostic efficacy of these methods was explored. The combination of LBSC and Boao MTB TaqMan-qPCR resulted in a positive rate of 54.4% (68 patients), representing increases of 20.8% and 5.6% compared to the respective rates of LBSC and Boao MTB TaqMan-qPCR alone. When GeneXpert MTB/RIF was added to the aforementioned method, the positive rate rose to 61.6%. Ultimately, the combination of all four testing methods achieved a positive rate of 68.8%.

**Fig. 3:**
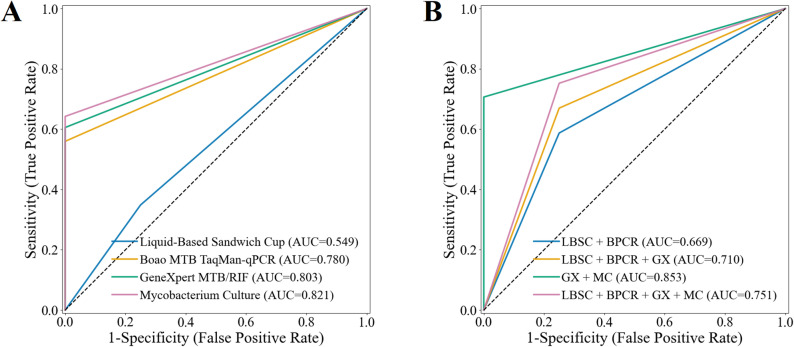
Receiver operating characteristic curves and areas under the curve of multiple testing methods on sputum specimens. (**A**) Performance of individual testing methods. (**B**) Performance of combined testing methods.

**Fig. 4:**
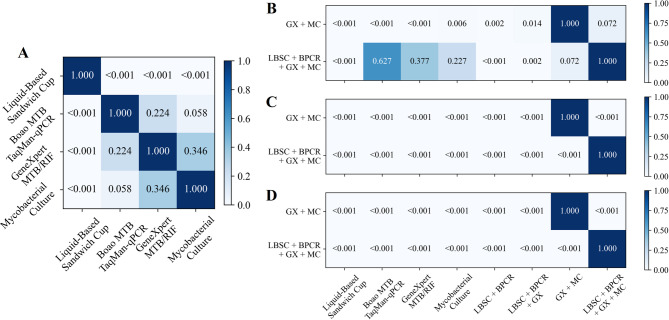
Comparison of P-values among individual and combined methods on sputum specimens. (**A**) Pairwise comparison of AUROC values for the four individual diagnostic methods. (**B**,** C** and **D**) Pairwise comparison of AUROC, Sensitivity and NPV values between combined methods and individual methods.

The diagnostic performance of the four sputum specimen testing methods was assessed in Table 2; Fig. 3A. Mycobacterial culture demonstrated the highest sensitivity and NPV, achieving 64.2% (95% CI 54.8–73.5%) and 29.1% (95% CI 18.0–41.3%), respectively, whereas LBSC exhibited the lowest sensitivity and NPV, at 34.9% (95% CI 26.1–43.4%) and 14.5% (95% CI 7.0–22.2%), respectively. Boao MTB TaqMan-qPCR, GeneXpert MTB/RIF, and mycobacterial culture all achieved a specificity and PPV of 100.0% (95% CI 100.0–100.0%). The AUROC for mycobacterial culture was 0.821 (95% CI 0.774–0.867), which was significantly higher than that of LBSC (*p* < 0.001), but not significantly different from GeneXpert MTB/RIF (0.803, 95% CI 0.757–0.848) or Boao MTB TaqMan-qPCR (0.780, 95% CI 0.731–0.826) (all *p* > 0.05 in Fig. 4A). In the analysis of combined methods for sputum specimens (Table 2; Fig. 3B), the combination of all four techniques yielded the highest sensitivity of 75.2% (95% CI 66.4–82.4%), significantly surpassing each individual method (Fig. 4B, all *p* < 0.001). Its NPV (30.8%) was comparable to that of the GeneXpert MTB/RIF plus mycobacterial culture combination (33.3%), with no significant difference between the two (*p* > 0.05). Notably, the GeneXpert MTB/RIF plus culture combination achieved the highest AUROC (0.853, 95% CI 0.808–0.894) compared with the four-method combination, as well as significantly higher specificity and PPV (both 100.0%, 95% CI 100.0–100.0%, all *p* < 0.05) in Fig. 4B-D.

**Table 2:** Diagnostic performance of multiple testing methods on sputum specimens.

Liquid-Based Sandwich Cup	AUROC	Sensitivity (%)	Specificity (%)	PPV (%)	NPV (%)
	0.549 [0.408, 0.662]	34.9 [26.1, 43.4]	75.0 [50.0, 94.7]	90.5 [80.0, 98.0]	14.5 [7.0, 22.2]
Boao MTB TaqMan-qPCR	0.780 [0.731, 0.826]	56.0 [46.2, 65.2]	100.0 [100.0, 100.0]	100.0 [100.0, 100.0]	25.0 [15.1, 37.5]
GeneXpert MTB/RIF	0.803 [0.757, 0.848]	60.6 [51.4, 69.5]	100.0 [100.0, 100.0]	100.0 [100.0, 100.0]	27.1 [15.7, 38.6]
Mycobacterial Culture	0.821 [0.774, 0.867]	64.2 [54.8, 73.5]	100.0 [100.0, 100.0]	100.0 [100.0, 100.0]	29.1 [18.0, 41.3]
LBSC + BPCR	0.669 [0.533, 0.777]	58.7 [50.0, 67.6]	75.0 [50.0, 94.1]	94.1 [87.3, 98.6]	21.1 [10.9, 32.1]
LBSC + BPCR + GX	0.710 [0.596, 0.816]	67.0 [57.8, 75.7]	75.0 [52.9, 93.8]	94.8 [89.3, 98.8]	25.0 [14.0, 37.8]
GX + MC	0.853 [0.808, 0.894]	70.6 [61.7, 78.8]	100.0 [100.0, 100.0]	100.0 [100.0, 100.0]	33.3 [21.3, 46.7]
LBSC + BPCR + GX + MC	0.751 [0.633, 0.867]	75.2 [66.4, 82.4]	75.0 [53.3, 95.0]	95.3 [90.9, 99.0]	30.8 [16.7, 45.5]

### Clinical performance by combining multiple testing methods on BALF specimens

**Fig. 5:**
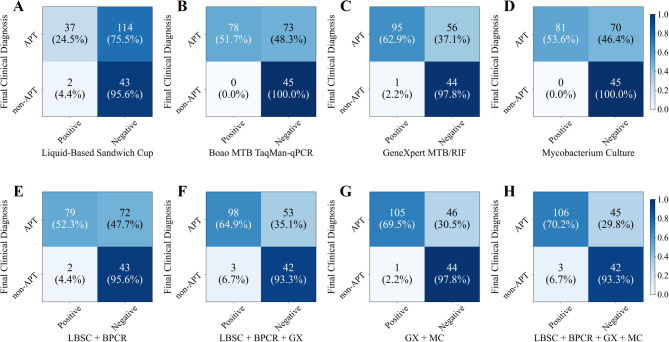
Confusion matrix of multiple testing methods on BALF specimens.

For the analysis of BALF specimens, 196 patients were selected from the study population, each undergoing LBSC, Boao MTB TaqMan-qPCR, GeneXpert MTB/RIF and mycobacterial culture. 39 patients (19.8%) achieved a positive LBSC, 78 patients (39.8%) achieved a positive Boao MTB TaqMan-qPCR, 96 patients (49.0%) achieved a positive GeneXpert MTB/RIF, and 81 patients (41.3%) achieved a positive mycobacterial culture (Fig. 5). Furthermore, a higher positive rate can be obtained by combining multiple testing methods and the combination of all four testing methods resulted in the highest positive rate of 55.6% (109 patients). The combination of GeneXpert MTB/RIF and mycobacterial culture resulted in the second-highest positive rate of 54.1% (106 patients), representing increases of 12.8% compared to the respective rates of the combination of LBSC and Boao MTB TaqMan-qPCR.

**Fig. 6:**
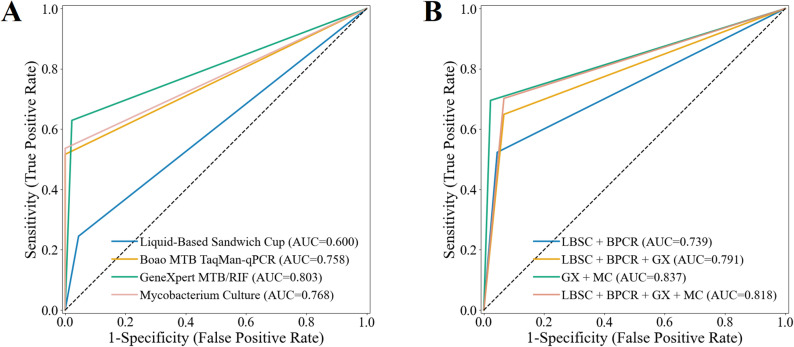
Receiver operating characteristic curves and areas under the curve of multiple methods on BALF specimens. (**A**) Performance of individual testing methods. (**B**) Performance of combined testing methods.

**Fig. 7:**
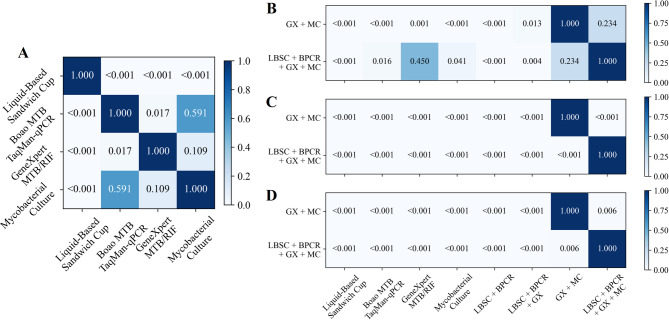
Comparison of P-values among individual and combined methods on BALF specimens. (**A**) Pairwise comparison of AUROC values for the four individual diagnostic methods. (**B**, **C** and **D**) Pairwise comparison of AUROC, Sensitivity and NPV values between combined methods and individual methods.

The performance of the four BALF specimen testing methods was evaluated as shown in Table 3; Figs. 6A and 7A. GeneXpert MTB/RIF demonstrated the highest performance among single methods in AUROC, sensitivity, and NPV, achieving 0.803 (95% CI 0.759–0.847), 62.9% (95% CI 55.1–70.4%), and 44.0% (95% CI 34.9–53.6%), respectively. Boao MTB TaqMan-qPCR and mycobacterial culture achieved a specificity and positive predictive value (PPV) of 100.0% (95% CI 100.0–100.0%). However, due to one false-positive case with GeneXpert MTB/RIF, its specificity and PPV were slightly lower, at 97.8% (95% CI 92.3–100.0%) and 99.0% (95% CI 96.7–100.0%), respectively. As shown in Table 3; Fig. 6B, the combination of all four testing methods yielded the highest sensitivity (70.2%, 95% CI 63.3–77.4%). This value was higher than those of the individual methods, the difference from GeneXpert alone (62.9%, 95% CI 55.1–70.4%) and from the GeneXpert plus culture combination (69.5%, 95% CI 61.6–77.3%) was significant (*p* < 0.05, Fig. 7C). Notably, the GeneXpert plus culture combination achieved the highest NPV (48.9, 95% CI 38.2–58.9%) higher than most other strategies (*p* < 0.05, Fig. 7D) and the highest AUROC (0.837, 95% CI 0.789–0.878, Fig. 7B), without significance with the four-method combination.

**Table 3:** Diagnostic performance of multiple methods on BALF specimens.

Liquid-Based Sandwich Cup	AUROC	Sensitivity (%)	Specificity (%)	PPV (%)	NPV (%)
	0.600 [0.551, 0.647]	24.5 [18.1, 31.7]	95.6 [89.1, 100.0]	94.9 [87.0, 100.0]	27.4 [20.3, 34.0]
Boao MTB TaqMan-qPCR	0.758 [0.720, 0.796]	51.7 [44.0, 59.2]	100.0 [100.0, 100.0]	100.0 [100.0, 100.0]	38.1 [29.2, 47.2]
GeneXpert MTB/RIF	0.803 [0.759, 0.847]	62.9 [55.1, 70.4]	97.8 [92.3, 100.0]	99.0 [96.7, 100.0]	44.0 [34.9, 53.6]
Mycobacterial Culture	0.768 [0.730, 0.809]	53.6 [46.0, 61.8]	100.0 [100.0, 100.0]	100.0 [100.0, 100.0]	39.1 [30.6, 48.7]
LBSC + BPCR	0.739 [0.687, 0.786]	52.3 [44.4, 60.1]	95.6 [88.7, 100.0]	97.5 [93.5, 100.0]	37.4 [28.6, 46.1]
LBSC + BPCR + GX	0.791 [0.736, 0.842]	64.9 [56.9, 72.3]	93.3 [85.2, 100.0]	97.0 [93.3, 100.0]	44.2 [34.8, 54.8]
GX + MC	0.837 [0.789, 0.878]	69.5 [61.6, 77.3]	97.8 [93.0, 100.0]	99.1 [96.9, 100.0]	48.9 [38.2, 58.9]
LBSC + BPCR + GX + MC	0.818 [0.764, 0.871]	70.2 [63.3, 77.4]	93.3 [85.4, 100.0]	97.2 [93.9, 100.0]	48.3 [37.6, 59.8]

### Clinical performance by combining multiple test methods and multiple specimens

**Fig. 8:**
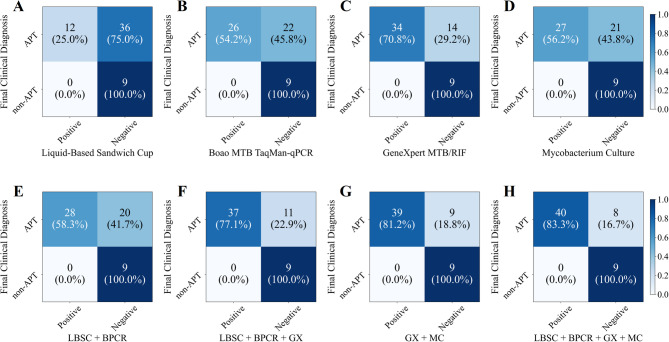
Confusion matrix of multiple testing methods on sputum and BALF specimens.

The above two sets of experiments conducted diagnostic analyses on sputum and BALF specimens, utilizing both individual and combined testing methods, including LBSC, Boao MTB TaqMan-qPCR, GeneXpert MTB/RIF and mycobacterial culture. To further validate the performance of the combined testing methods, 57 patients were selected from the study population, each undergoing 4 testing methods on both sputum and BALF specimens. Among the four testing methods, the combination of sputum and BALF specimens revealed that GeneXpert MTB/RIF identified 34 patients (59.6%) as positive, surpassing the mycobacterial culture tested 27 positive patients (47.4%), Boao MTB TaqMan-qPCR tested 26 positive patients (45.6%), and LBSC tested 12 positive patients (21.1%). Furthermore, when combining LBSC and Boao MTB TaqMan-qPCR, 28 patients (49.1%) tested positive, and the inclusion of GeneXpert MTB/RIF further increased the positive rate to 37 patients (64.9%). The combination of all four test methods yielded the highest number of positive cases, identifying 40 patients (70.2%) as positive. Notably, the combination of GeneXpert MTB/RIF and mycobacterial culture was only one patient short of the total detected by all four methods (Fig. 8).

**Fig. 9:**
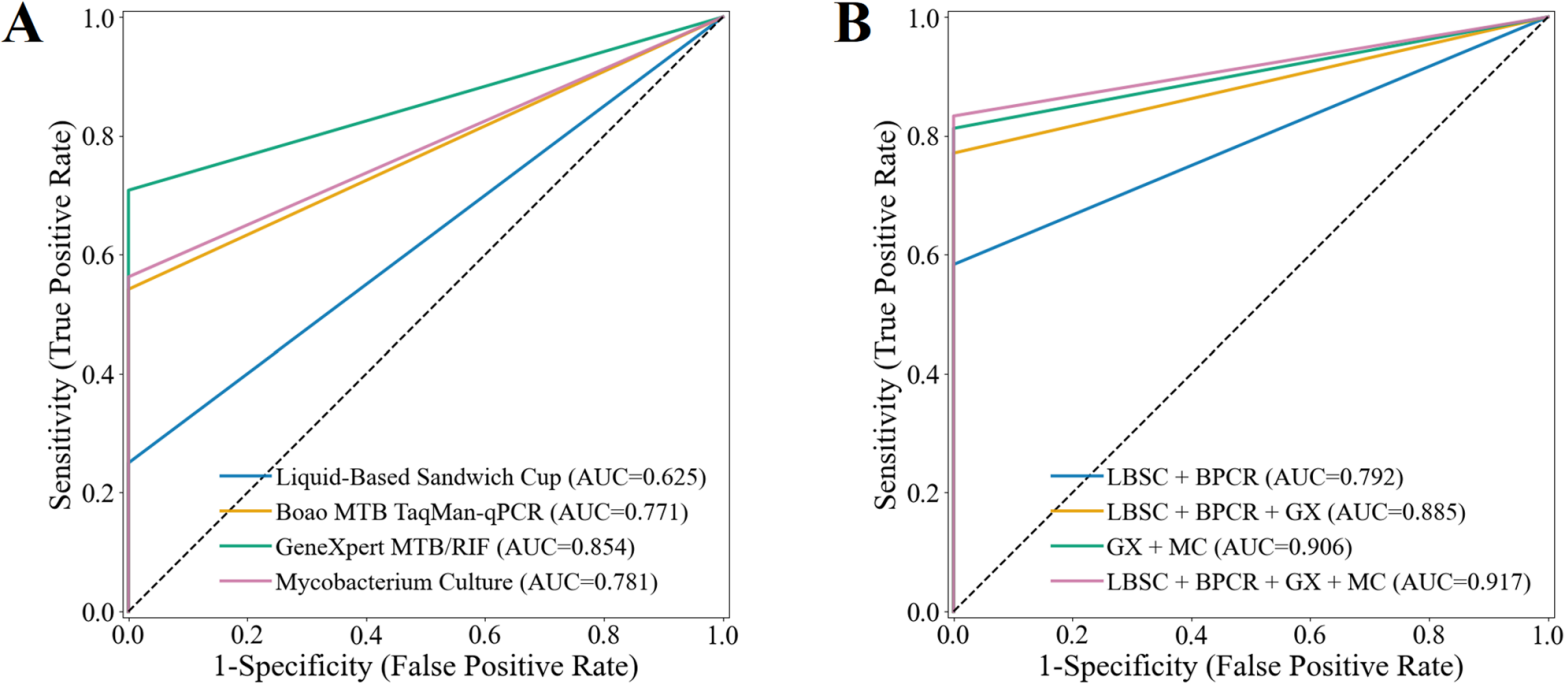
Receiver operating characteristic curves and areas under the curve of multiple methods on sputum and BALF specimens. (**A**) Performance of individual testing methods. (**B**) Performance of combined testing methods.

**Fig. 10:**
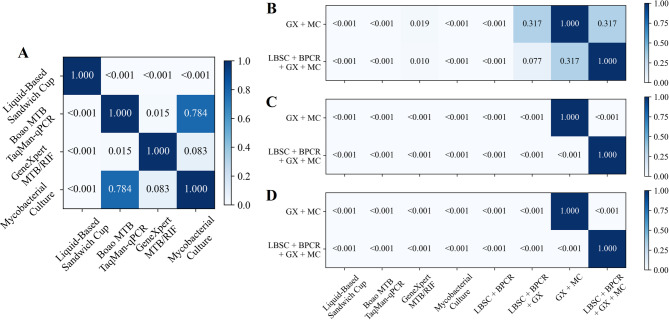
Comparison of P-values among individual and combined methods on sputum and BALF specimens. (**A**) Pairwise comparison of AUROC values for the four individual diagnostic methods. (**B**, **C** and **D**) Pairwise comparison of AUROC, Sensitivity and NPV values between combined methods and individual methods.

Based on the final clinical diagnosis, we evaluated the diagnostic performance of four testing methods, combining sputum and BALF specimens. In the analysis of individual testing methods (Table 4; Figs. 9 and 10A), GeneXpert MTB/RIF demonstrated the best performance, with an AUROC of 0.854 (95% CI 0.788–0.917), sensitivity of 70.8% (95% CI 57.7–83.3%), specificity of 100.0% (95% CI 100.0-100.0.0.0%), PPV of 100.0% (95% CI 100.0-100.0.0.0%), and NPV of 39.1% (95% CI 19.2–60.0%). mycobacterial culture and Boao MTB TaqMan-qPCR demonstrated comparable diagnostic performance. LBSC exhibited the poorest results, with an AUROC of 0.625 (95% CI 0.567–0.693), sensitivity of 25.0% (95% CI 13.5–38.6%), and NPV of 20.0% (95% CI 9.3–33.3%). The combination of multiple test methods significantly improved diagnostic performance. The combination of all four testing methods achieved the optimal performance, with an AUROC of 0.917 (95% CI 0.860–0.968), sensitivity of 83.3% (95% CI 72.0–93.6.0.6%), and NPV of 52.9% (95% CI 26.7–78.6%), substantially outperforming the individual testing methods (Fig. 10B-D). The combination of GeneXpert MTB/RIF and mycobacterial culture yielded the second-best results, with an AUROC of 0.906 (95% CI 0.848–0.958). This AUROC did not show a statistically significant difference compared to the four-method combination (Fig. 10B, *p* = 0.317). However, there was a statistically significant difference in sensitivity (Fig. 10C, *p* < 0.001). Interestingly, all methods achieved a specificity and PPV of 100.0% (95% CI 100.0-100.0.0.0%).

**Table 4:** Diagnostic performance of multiple methods on sputum and BALF specimens.

Liquid-Based Sandwich Cup	AUROC	Sensitivity (%)	Specificity (%)	PPV (%)	NPV (%)
	0.625 [0.567, 0.693]	25.0 [13.5, 38.6]	100.0 [100.0, 100.0]	100.0 [100.0, 100.0]	20.0 [9.3, 33.3]
Boao MTB TaqMan-qPCR	0.771 [0.704, 0.837]	54.2 [40.8, 67.4]	100.0 [100.0, 100.0]	100.0 [100.0, 100.0]	29.0 [12.1, 45.8]
GeneXpert MTB/RIF	0.854 [0.788, 0.917]	70.8 [57.7, 83.3]	100.0 [100.0, 100.0]	100.0 [100.0, 100.0]	39.1 [19.2, 60.0]
Mycobacterial Culture	0.781 [0.706, 0.850]	56.2 [41.2, 70.0]	100.0 [100.0, 100.0]	100.0 [100.0, 100.0]	30.0 [13.6, 46.7]
LBSC + BPCR	0.792 [0.722, 0.857]	58.3 [44.4, 71.4]	100.0 [100.0, 100.0]	100.0 [100.0, 100.0]	31.0 [15.6, 48.0]
LBSC + BPCR + GX	0.885 [0.824, 0.942]	77.1 [64.7, 88.4]	100.0 [100.0, 100.0]	100.0 [100.0, 100.0]	45.0 [25.0, 69.6]
GX + MC	0.906 [0.848, 0.958]	81.2 [69.6, 91.7]	100.0 [100.0, 100.0]	100.0 [100.0, 100.0]	50.0 [26.1, 73.7]
LBSC + BPCR + GX + MC	0.917 [0.860, 0.968]	83.3 [72.0, 93.6]	100.0 [100.0, 100.0]	100.0 [100.0, 100.0]	52.9 [26.7, 78.6]

## Discussion

Tuberculosis is an infectious disease caused by *Mycobacterium* tuberculosis. The diagnosis of PTB is a multi-step process that typically requires the integration of various testing methods to ensure accurate identification and timely treatment. Phenotypic tests, including LBSC and mycobacterial culture, alongside molecular biological tests such as Boao MTB TaqMan-qPCR and GeneXpert MTB/RIF, are widely employed in the diagnostic process for presumed PTB. This study aims to evaluate the diagnostic value of combining phenotypic and molecular biological methods in the identification of PTB, as well as to assess the diagnostic performance of different specimen types (sputum and BALF) and their combinations.

We conducted a retrospective review and collected data from 264 presumed PTB patients with sputum or BALF specimens. According to the national diagnostic standards (WS 288–2017 for tuberculosis diagnosis), 212 patients were confirmed to have APT, while the remaining 52 patients were diagnosed as non-APT. To analyze the diagnostic efficacy of combining phenotypic and molecular biological methods, we established three experimental groups for data selection: (A) 125 patients who underwent LBSC, Boao MTB TaqMan-qPCR, GeneXpert MTB/RIF and mycobacterial culture on sputum specimens, (B) 196 patients who underwent the same tests on BALF specimens, and (C) 57 patients who underwent the same tests on both sputum and BALF specimens.

The combination of multiple testing methods significantly enhances the positive rate and achieves higher sensitivity in diagnosis. The combination of all four tests yields the highest sensitivity in both sputum and BALF specimens, with significant improvements compared to other individual or combined testing methods (all *p* < 0.001 in Figs. 4C and 7C, and 10C). Additionally, the combination of GeneXpert MTB/RIF and mycobacterial culture demonstrates better AUROC, achieving 0.853 (95% CI 0.808–0.894) for sputum specimens and 0.803 (95% CI 0.759–0.847) for BALF specimens. This is due to the high positive rates from the four-test combination, which may lead to overdiagnosis, misclassifying some non-APT cases as APT. This affects specificity and subsequently reduces overall classification performance. In contrast, no overdiagnosis was observed in the combined analyses of sputum and BALF specimens due to the low number of non-APT cases. While the AUROC for the four-test combination is slightly higher, there was no significant difference between the four-test combination and the combination of GeneXpert MTB/RIF and mycobacterial culture in terms of AUROC across the three specimen types (*p* = 0.072 in Fig. 4A, *p* = 0.234 in Fig. 7A, and *p* = 0.317 in Fig. 10A).

It’s worth noting that the sensitivity of various testing methods (both individual and combined methods) on BALF specimens was lower compared to sputum specimens in this study. Among 264 enrolled patients, 143 had sputum specimens tested with GeneXpert MTB/RIF. Of these, 65 were negative and 78 were positive. Of the 65 negative sputum cases, 49 underwent GeneXpert MTB/RIF of BALF specimens, yielding a submission rate of 75.38%. Among the 78 sputum-positive cases, only 18 underwent GeneXpert MTB/RIF of BALF specimens, with a submission rate of 23.08%. However, 264 enrolled patients had BALF specimens tested with GeneXpert MTB/RIF. Of these, 49 cases were sputum GeneXpert-negative, 136 had little or no sputum, and 18 were GeneXpert-positive on sputum specimens. This means that 91.13% of cases who underwent GeneXpert MTB/RIF of BALF specimens were either sputum-negative or had little to no sputum. In clinical practice, when sputum is abundant, physicians tend to prioritize sputum testing, and patients with sufficient sputum are typically those with more extensive or severe pulmonary lesions, often correlating with higher bacterial loads. For patients with little or no sputum, or those with negative sputum results, clinicians are more likely to consider bronchoscopy, which is usually performed on patients with milder or more localized pulmonary disease and lower bacterial loads. As this study was based on real-world clinical data, the lower sensitivity of both single and combined bronchoscopic tests compared to sputum testing reflects this clinical practice pattern. Furthermore, considering the higher cost of GeneXpert (The hospital provides only one free testing session), clinicians are more selective in submitting samples for testing. This selectivity is not only to avoid unnecessary costs, but also to ensure that the submitted specimens are clinically appropriate (e.g., obtained from patients with a reasonable pre-test probability of tuberculosis). Such practice helps maintain diagnostic accuracy by minimizing low-yield or inappropriate testing, which can otherwise compromise laboratory efficiency and increase the likelihood of false negatives or inconclusive results.

**Fig. 11:**
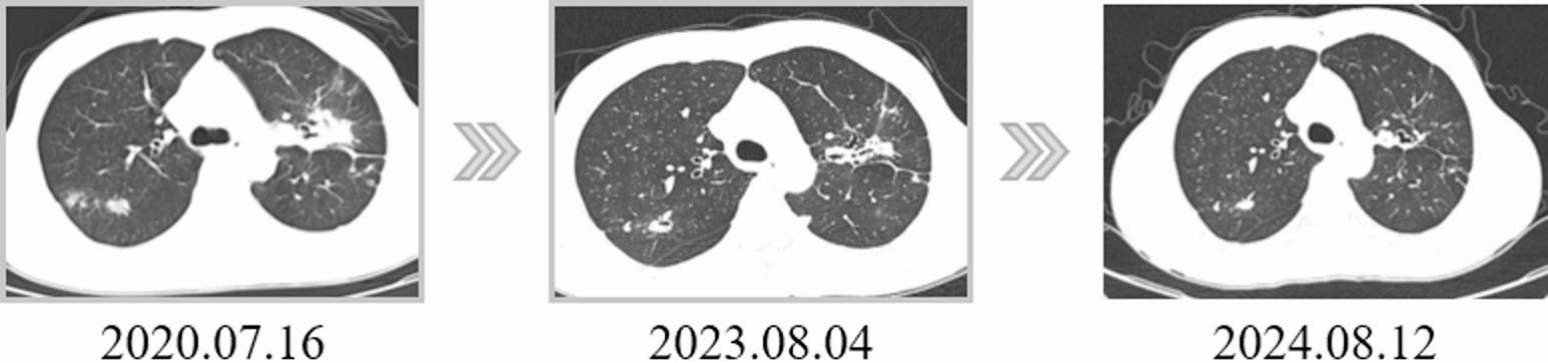
Chest CT of cases where non-APT was misclassified as APT using GeneXpert MTB/RIF on BALF specimens.

The specificity and PPV of GeneXpert MTB/RIF on BALF specimens were 97.8% (95% CI: 92.3–100.0%) and 99.0% (95% CI: 96.7–100.0%), due to only one case of inactive PTB classified as APT. In this case, the patient had a positive GeneXpert MTB/RIF result on BALF specimens with low bacterial load, detected rifampicin resistance, but was ultimately diagnosed with non-APT. The patient was re-admitted due to chest discomfort after 3 days, and both sputum and BALF tests (LBSC and Boao MTB TaqMan-qPCR) were negative. The patient had a prior diagnosis of drug-resistant tuberculosis and had undergone a standard regimen of 4AmLfxPtoCsLzdE/2LfxCsLzd/18LfxCsCfzZE for 24 months. A follow-up chest CT upon admission showed bilateral lung lesions with consolidation, which had been absorbed compared to previous imaging, with no new lesions. A small cavity was observed in the right upper lobe, thought to be a post-granuloma liquefaction cavity. Forty-two days later, mycobacterial cultures of both sputum and BALF specimens were negative, and follow-up after one year revealed that the purified cavity had spontaneously closed, and bilateral lung lesions continued to heal naturally in Fig. 11. Although GeneXpert MTB/RIF is highly sensitive and specific, its DNA-based test cannot distinguish between live and dead bacteria, and DNA fragments may persist in the body for a long time due to their stable structure. Despite the positive GeneXpert MTB/RIF, a combination of clinical imaging findings and patient’s treatment history suggested a diagnosis of non-APT. Subsequent negative cultures and imaging follow-up further confirmed this diagnosis. In clinical practice, the specificity and PPV of GeneXpert MTB/RIF for APT are often lower than the findings of this study. Our study cohort included relatively few patients with a history of PTB, focusing primarily on those presumed of APT. As a result, cases ultimately diagnosed as inactive PTB were classified in the non-APT group. Our study was to assess the diagnostic performance of GeneXpert MTB/RIF for PTB, not to determine disease activity. Therefore, while a positive GeneXpert MTB/RIF confirms tuberculosis, it does not necessarily indicate active tuberculosis, and GeneXpert MTB/RIF is not recommended as a tool for monitoring treatment efficacy.

Our study also compared the performance of the combination of GeneXpert MTB/RIF (GX) and mycobacterial culture (MC) against the combined use of all four diagnostic methods (LBSC, BPCR, GX, and MC). While the four-method combination achieved the highest sensitivity of 83.3%, it is noteworthy that the sensitivity difference between GX + MC (81.2%) and LBSC+ BPCR + GX+MC (83.3%) was not statistically significant, as indicated by the overlapping 95% confidence intervals. The combination of GX + MC showed a comparable sensitivity and a slightly higher NPV (33.3%) compared to the four-method combination (30.8%), but the differences in these parameters did not reach statistical significance. However, the combination of GX + MC demonstrated a significantly higher AUROC (0.853) compared to the four-method combination (0.818), with a marked improvement in specificity and PPV (both 100.0%). These results suggest that while the four-method combination enhances sensitivity, GX + MC may offer better overall diagnostic accuracy, particularly in terms of specificity and positive predictive value. This finding is consistent with our clinical observation that GX + MC, as a more targeted approach, may reduce overdiagnosis by identifying patients with higher bacterial loads or more active disease. Therefore, while the four-method combination is beneficial in increasing sensitivity, GX + MC provides a more accurate diagnostic result in terms of specificity and PPV, which may be particularly important for distinguishing active from non-active TB cases. Future studies with larger sample sizes are needed to better understand the trade-offs between sensitivity and specificity, and to explore whether GX + MC should be prioritized in certain clinical settings, especially in resource-limited environments.

Our findings also raise important considerations regarding the diagnostic strategy of applying multiple methods to the same specimen and testing multiple specimens from the same patient. Performing several assays on a single sample can enhance sensitivity and provide complementary information, yet it may also lead to discordant results that complicate clinical interpretation and increase laboratory costs. Similarly, testing multiple specimens, such as sputum and BALF or repeated sputum collections, improves the likelihood of detecting patients with low bacterial burden, but this approach inevitably increases resource utilization. From a public health perspective, the balance between diagnostic yield and cost-effectiveness must therefore be carefully evaluated.

In China, a high-burden country for tuberculosis, the government has provided unprecedented support for TB control through public health authorities. For presumed and clinically diagnosed TB patients, GeneXpert testing is currently offered free of charge in our hospital (only one free testing session), while conventional methods such as acid-fast staining, mycobacterial culture, and melting curve-based fluorescence PCR are all covered by the basic medical insurance scheme. Although the combined use of these diagnostic methods may appear costly, this approach enables earlier and more accurate diagnosis and treatment, which helps to prevent delays in care, disease progression, and further transmission. From a public health perspective, such investment is justified and cost-effective, aligning with the World Health Organization’s goals for TB control.

Our study was limited by the number of patients included, and not all patients underwent LBSC, Boao MTB TaqMan-qPCR, GeneXpert MTB/RIF and mycobacterial culture on both sputum and BALF specimens. Only 57 patients underwent both specimen types and all four tests, of which only 9 were diagnosed as non-APT. In this study, the four-test combination resulted in all 9 non-APT cases being classified as negative, yielding a specificity and PPV of 100%. The low number of negative specimens may affect the fairness of the diagnostic classification performance.

## Conclusion

This study demonstrates that the combination of phenotypic and molecular biological tests, along with an expanded range of specimen types, significantly enhances the positive detection rate of pulmonary tuberculosis. This approach offers high sensitivity and comparable classification performance, thereby providing more effective guidance for treating PTB.

## Supplementary Information

Below is the link to the electronic supplementary material.

Supplementary Material 1

## Data Availability

All the data and material were true and available. The data is available from the corresponding author upon reasonable request.
