# Supplementary material for: Diagnostic value of phenotypic testing combined with molecular biology testing for tuberculosis
Source: Sci Rep. 2026 Mar 6;16:8692. doi: 10.1038/s41598-026-43218-z (PMC12979635; doi:10.1038/s41598-026-43218-z)
Supplement: Supplementary file 1 — Supplementary Material 1 [file 41598_2026_43218_MOESM1_ESM.docx]

**Section S1: Test methods**

***Phenotypic Test Methods:***

(1) Liquid-based sandwich cup acid-fast staining: Specimens were first treated with a standard digestive solution to liquefy mucus and reduce contamination. The processed suspension was filtered through a specialized sandwich cup equipped with a nanostructured silica membrane, which concentrates acid-fast bacilli onto the membrane surface. The membrane was subsequently stained using the Ziehl-Neelsen method and examined under a light microscope by trained personnel. The enrichment process significantly improves the sensitivity of smear microscopy compared with conventional direct smears. The detail procedure of liquid-based sandwich cup acid-fast staining is shown in Fig. S1.

(2) Mycobacterial culture: Specimens were inoculated into the BD BACTEC MGIT 960 liquid culture system (Becton Dickinson, USA), which continuously monitors oxygen consumption via fluorescence quenching to detect mycobacterial growth. The system provides automated, real-time assessment of culture positivity and shortens the turnaround time compared with solid media. Positive signals were confirmed by Ziehl–Neelsen staining for acid-fast bacilli, and isolates were further identified to distinguish Mycobacterium tuberculosis complex from nontuberculous mycobacteria when necessary. Contaminated cultures were excluded after subculture on blood or chocolate agar to check for bacterial overgrowth.

***Molecular Biology Test Methods:***

(1) Boao MTB TaqMan-qPCR: This assay is performed using a commercial Tuberculosis/Nontuberculous Mycobacterium Nucleic Acid Detection Kit (Chengdu Boao Jingxin Biotechnology Co., Ltd.), which is based on a TaqMan probe-based real-time PCR assay. This method employs dual PCR amplification with specific primers and fluorescently labeled TaqMan probes targeting the Mycobacterium tuberculosis complex and nontuberculous mycobacteria. Fluorescence signals are monitored in different channels during amplification to determine the presence of target genes. According to the manufacturer’s instructions, nucleic acids were extracted and amplified on a real-time PCR system (CFX96 Deep Well). A sigmoidal amplification curve with Ct <40 in the FAM channel was interpreted as positive for M. tuberculosis complex, whereas a positive sigmoidal curve in the HEX channel with no amplification in FAM indicated nontuberculous mycobacteria. Samples without amplification curves in either channel were considered negative.

(2) GeneXpert MTB/RIF: This assay is a fully automated, cartridge-based nucleic acid amplification test that enables rapid detection of Mycobacterium tuberculosis and rifampicin resistance. The assay integrates four steps—specimen processing, DNA extraction, real-time PCR amplification, and fluorescent probe-based detection—within a single disposable cartridge. The system targets the rpoB gene hotspot region, allowing simultaneous identification of M. tuberculosis and the most common mutations associated with rifampicin resistance. Results are available within two hours, with minimal hands-on time, making this method suitable for routine clinical and point-of-care testing.


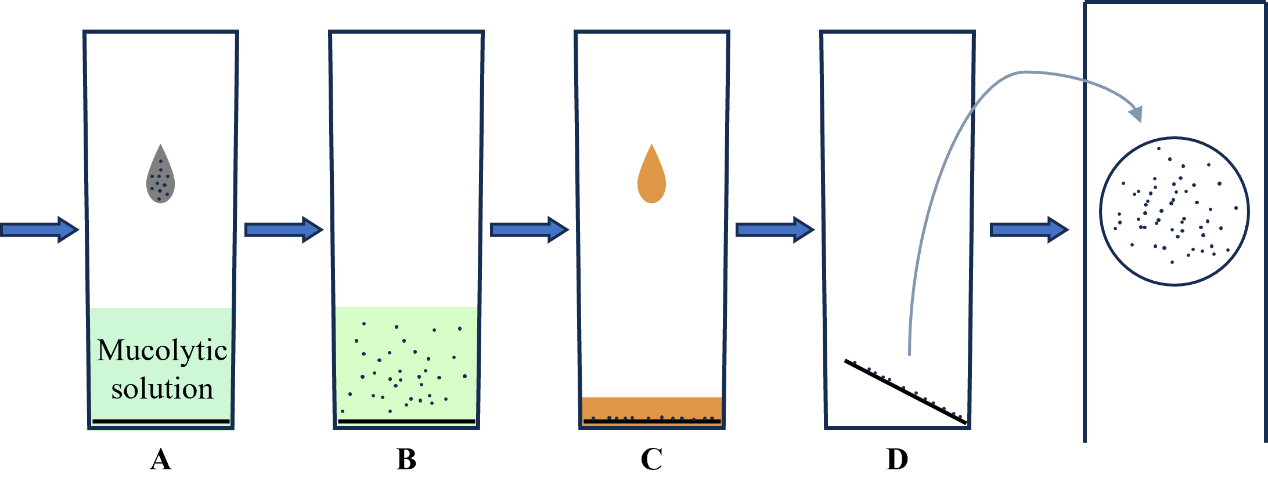


**Fig. S1** Procedure of Liquid-based sandwich cup acid-fast staining. (A) Clinical specimens were directly collected into the sandwich cup. (B) A dedicated centrifuge was used to sediment mycobacteria onto the basal membrane of the cup. (C) Heat fixation and Ziehl-Neelsen staining were performed directly within the cup. (D) The membrane was mounted on a slide, sealed with a coverslip, and examined microscopically under biosafety conditions.

**Section S2: Patients and study collective characteristics**

Further comparing the characteristics of patients diagnosed with APT and non-APT, the ratio of APT patients diagnosed with diabetes mellitus (18.4%) was higher than those of non-APT patients (13.5%) without statistically significant (*p* = 0.524). Besides, the proportions of cough (74.1%) or expectoration (57.5%) symptoms were higher in APT patients compared to non-APT patients (63.5%, 44.2%). Similarly, APT patients exhibited a greater incidence of effusion (45.3%) and calcification (50.0%) on CT imaging than non-APT patients (34.6%, 42.3%); however, these differences were not statistically significant (*p*=0.176 for cough, *p*=0.116 for expectoration, *p*=0.217 for effusion, *p*=0.401 for calcification). However, the age of APT patients was significantly smaller than that of non-APT patients (44 [26, 63] vs. 58 [46, 66], *p*=0.022).
